# Racing Against Time Against Zika

**DOI:** 10.4269/ajtmh.16-0563

**Published:** 2016-08-03

**Authors:** Claire Panosian Dunavan

**Affiliations:** ^1^ University of California, Los Angeles, CA. E-mail: cpanosian@mednet.ucla.edu

**Figure 1:**
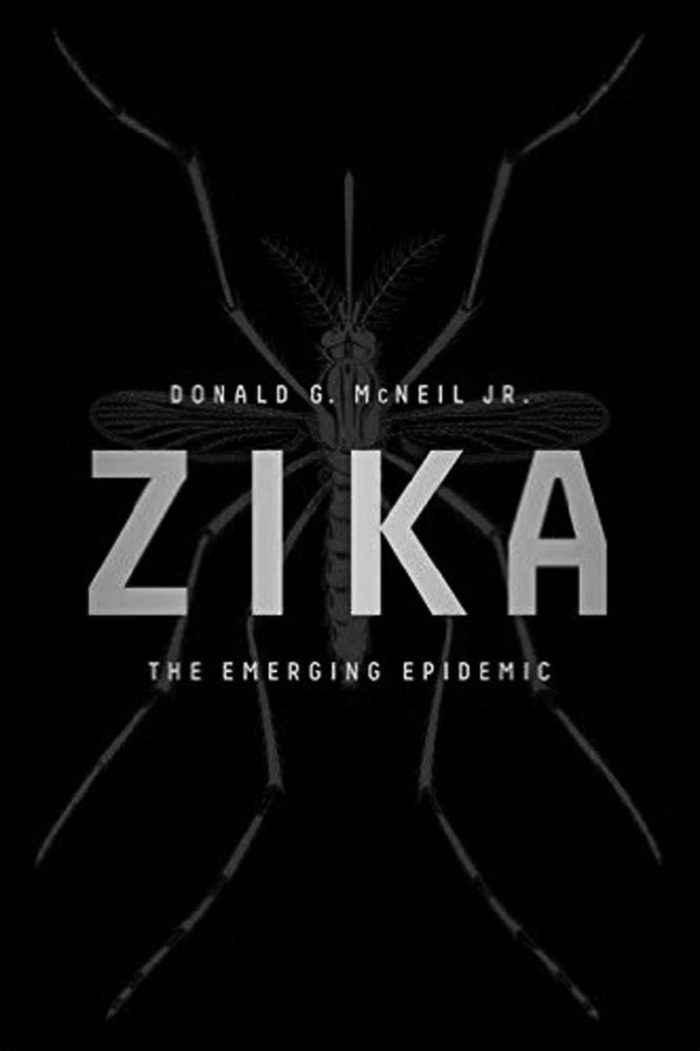

Eight thousand microcephalic babies born, and many more to come. Seven months and counting: still no congressional response. The Olympics in Rio just around the corner. And overshadowing it all: millions of women, men, and families in 50 countries and territories living in fear.

How long the fear will continue is hard to say. But at the time of this writing, time itself is a major actor in the drama of Zika. The time it will take for the tropical virus that first emerged in Uganda to circle the globe. The time required for a female *Aedes* to breed, “sip,” and infect. The time a pregnant woman must wait to learn (today? tomorrow? in 5 or 10 years?) whether her child is in some way maimed. The time needed to make a Zika vaccine—or a new insecticide—or to successfully release a genetically modified mosquito.

Last spring, while time was working in Zika's favor, it was also dictating the life of Donald G. McNeil Jr., a *New York Times* reporter writing about the crisis. Feverishly typing into the night, McNeil had 30 days to complete a book. On May 26, he submitted his draft. On July 5, *Zika-The Emerging Epidemic* hit Amazon trucks and airport newsstands.

Years from now, wherever we stand in the struggle, many of us will still be reading and sharing McNeil's real-time account of Zika's stunning assault on an unprepared planet. Yes, the book is really that good.

What public health authorities should have told—and should still be telling—women of child-bearing age in Zika-afflicted sites is, not surprisingly, a key question in McNeil's fast-paced narrative. Consider the following excerpt from a chapter entitled “Delaying pregnancy”:

The CDC and WHO continued issuing the same advice: avoid mosquito bites. Use DEET, wear long sleeves. Hold tight while we work on a vaccine.

Off the record, however, people in the CDC and its overseer, the Department of Health and Human Services, told me the issue was splitting their agencies. It had become, they said, a debate between two camps: the infectious diseases specialists, who felt that asking women to delay was the only way to save their babies, versus the reproductive health specialists, who said the government should not tell women what to do with their bodies.

But no one in the infectious disease camp would be quoted disagreeing with CDC policy.

A few days later, the World Health Organization (WHO) echoed the Centers for Disease Control and Prevention's (CDC's) verdict, namely that pregnant travelers should simply stay home rather than risk infection in stricken locales. Back to the journalist:

During the telephone conference afterward, I asked, not very politely, “If you're telling pregnant women not to visit countries with Zika because it's too dangerous, why aren't you telling women who live in those countries not to get pregnant? It seems inconsistent.

This is just a taste of the uncensored voice of McNeil's debut book; drawing on years spent covering other global scourges from malaria to acquired immunodeficiency syndrome (AIDS) to avian flu, sometimes he practically revels in challenging global health rhetoric. At the same time, readers will find the personal, medical, and scientific content of the book *Zika—The Emerging Epidemic* lucid, even prescient, especially given the book's lightning-fast birth.

Finally, beneath its crusty veneer, the book packs arresting images, “inconvenient truths,” and, above all, empathy.

The spray trucks featured in so much television footage from South America were largely useless publicity ploys. Governments liked them because people found them reassuring. But against Aedes aegypti mosquitoes, relying heavily on street fogging was almost counterproductive: they bred near houses and slipped indoors as soon as they could following the carbon dioxide vapor trail of human breath.

Hospital hallways, doctors remembered in Brazil, were lined with mothers who resembled ghosts. They were in shock: mute, expressionless, bleak. Some were just teenagers. Some had ridden buses for hours …. And there were so many of them. One doctor from southern Brazil, where there was no problem, recalled visiting a friend's hospital in Salvador, not at all expecting what he found: 25 babies with microcephaly, all born in the previous 10 days.

For many people—certainly many Americans—the scare may be brief: a vacation canceled, a business trip replaced by a phone call. For some, living in tropical climates, it will mean months of worry: Worry that each mosquito might be the dangerous one. Worry that they had a silent infection. For women who are pregnant, that worry might be sheer terror: having to ask themselves every day for nine months, “Is my baby all right? Was it my fault? Did I do everything I could to protect it?” … And a mother's worry does not end even on her deathbed: she may die wondering who will take care of the child for the rest of his or her life. Will those family caretakers have the money? Will they have the patience? Will they have the strength? And will they not hate her memory for leaving them the burden?

For a science correspondent, this is fearless prose indeed. So when will McNeil next forego sleep, race against time, and churn out another politically charged book about tropical medicine? Maybe he will tell us at November's annual meeting. But first, read this one.

## Five Questions for Donald G. McNeil Jr.

**For years, you have resisted writing a book. So why this book? And why did you write it in May 2016 as opposed to 6 months later, when scientists would have known so much more about Zika?**

I have resisted for several reasons: I dislike selling myself. I have watched *Times* colleagues take unpaid book leaves and slave away for months or years and then look crushed when their books are not bestsellers. Also, near my desk is a giveaway table full of science books that arrive in the mail, and we throw there unread. I did not want to prostrate myself to publishers, lose money, and end up on that table.

I wrote this one because Norton's editor-in-chief called in April and asked if I would write a book by May to hit bookstores in June. That was the deal: 30,000 words in 30 days or not a penny. That kind of assignment I can handle. I had no illusions that it would be “War and Peace.” I joked that it would be “Zika for Dummies,” but I hope it came out better than that.

**In *Zika-The Emerging Epidemic*, you have revealed rare, behind-the-scene conversations between journalists and global health leaders. Before you send an e-mail to, say, the chief spokesman for CDC Director Dr. Tom Frieden, do you consult with sources or editors, or simply go with your gut?**

Gut. If my editors had to vet my e-mails as well as my stories, I would never get anything done. Also, I do not usually need to be told what questions an average American wants answers to. (Just because I live in New York does not mean I cannot think like a real person.) When sources question the CDC's judgment, I ask the CDC for comment, of course. But sometimes I can spot weaknesses before they are pointed out to me; the needle on my BS meter starts to flicker.

**“One of the things we Americans do not realize is how truly indifferent many other governments are to the fates of their people” is one of the book's saddest comments. Please elaborate.**

That was in reference to the usefulness of the WHO as a wake-up call for some countries that a pathogenic threat is real. Most countries are governed by self-perpetuating elites. Most of the diseases I cover—AIDS, malaria, tuberculosis, polio, guinea worm, and all—hurt the folks at the opposite, powerless end of spectrum: the poorest poor, forest-dwelling tribes, sex workers, homosexuals, and all. Some diseases the elites ignore with impunity: no one in the capital scrambles the army to help the last village beat guinea worm. But when a disease is transmissible enough to threaten rich and poor alike, alarms get raised. The elites can still send their pregnant wives and daughters to London or Paris to avoid mosquitoes, but they cannot really do absolutely nothing for the poor or they would face riots or military coups. (Army officers sometimes come from rural villages and have some idea of how the poor suffer.)

Obviously, in the United States, we have relatively honest voting and pols eager to win reelection, so diseases do get fought, and sometimes the fights themselves create political power. The gay rights movement, the sexual revolution, and the fight against AIDS all evolved together; gay men united and are now a solid voting bloc in some American cities. But gay men in much of Africa are still literally hunted.

**What are your personal hopes and goals for this book?**

Well, to be completely selfish, I hope it sells, because I get a cut.

Beyond that, I hope two things: first, that it prods experts to openly debate a few questions like: Can mosquito control really protect pregnant women, or are we kidding ourselves? Should women delay pregnancy? Or—if that is too politically difficult for some officials—why are not we at least loudly reminding women that not being pregnant during peak transmission weeks is a surefire way to avoid birth defects? Also, why are we so squeamish about posters reminding people that they can get Zika from sex? I mean, we are not just talking about risks in a bathhouse, we are talking about married couples trying for babies. Sex is required—there is no reason to be shy about it.

For average Americans, including taxpayers, I hope the book at least hints that it makes sense to spend more money spotting epidemics early and having counterattacks ready, rather than having a media panic and a time-wasting fight in congress every time. When a new disease emerges, you want the CDC director to be able to go on TV and say “Relax. We have got this.” Instead, he has to go hat in hand to congress. If we had more vaccines ready to go; if we had more antiviral drugs; if we had better mosquito data; if we had more surveillance and laboratory capacity in Africa, Asia, and Latin America; if we had a national medical records system; if the WHO had a rapid reaction force, you name it, there are so many things that are better buys than wars in Iraq, ethanol subsidies or tax cuts for billionaires.

**When you first started your career as a copyboy for the *New York Times*, I am sure you never imagined you would be covering global infectious diseases. How has this beat changed you?**

It has made me much more empathic to the plight of the poor. I once slept in a pygmy hunting village in the Cameroonian jungle while doing a piece on bushmeat and AIDS—it was the kind of place where kids died for lack of a $1 measles shot or 50¢ worth of deworming medicine. And, because the only flight back to South Africa was via Paris, I found myself 48 hours later walking down the rue Saint Honoré looking at store windows full of men's suits costing $5,000. The unfairness of it all made me ill. This is not to bash the French—they are big donors to the Global Fund, they are pioneers in tropical medicine and AIDS medicine. And I am not particularly soft hearted: someone is always begging for money on my subway home to Brooklyn, I am usually cynically eyeballing their shoes or tattoos to see if I am really being asked to fund their crack habits. But large numbers of people in this world are well and truly screwed by circumstances that are in no way their fault. And they deserve help. From the American perspective, I think 50¢ to save the lives of their children is worth it. And, from a purely selfish point of view, that kind of foreign aid tends to make people love America rather than hating it.

